# Matters arising: In vivo effects of the alpha-synuclein misfolding inhibitor minzasolmin supports clinical development in Parkinson’s disease

**DOI:** 10.1038/s41531-024-00657-7

**Published:** 2024-03-14

**Authors:** Michel Alexander Steiner

**Affiliations:** Idorsia Pharmaceuticals Ltd, CNS Pharmacology, Hegenheimermattweg 91, 4123 Allschwil, Switzerland

**Keywords:** Diseases of the nervous system, Pharmacology, Neurophysiology, Neurological disorders, Psychiatric disorders

**arising from** D.L. Price et al. *npj Parkinson’s Disease* 10.1038/s41531-023-00552-7 (2023)

In their recent article, Diana L. Price and colleagues^1^ assessed the efficacy of the alpha-synuclein (aSyn) misfolding inhibitor minzasolmin (UCB0599) in a transgenic mouse model (Thy1-aSyn line 61) of Parkinson’s disease (PD). Minzasolmin is a drug candidate of UCB Pharma currently undergoing clinical testing for the treatment of PD in a phase II trial^2,3^. Chronic treatment with minzasolmin in the mouse PD model effectively reduced both behavioral deficits and protein markers of neuropathology. However, the authors did not discuss how the in vivo efficacy can be reconciled with minzasolmin’s low exposure in mice and a regularly interrupted treatment schedule^1^.

In the Thy1-aSyn mouse study, minzasolmin was administered for a total duration of three months by Monday to Friday daily (not Saturday and Sunday) intraperitoneal (IP) injection of a dose of 1 or 5 mg/kg. A prior, preparatory pharmacokinetic (PK) experiment in wild-type mice (C57BL/6) had revealed that minzasolmin readily entered the brain (total brain/plasma ratio of ~0.3) with a Tmax of 0.5 h and an almost linear dose-exposure relationship. At 1 and 5 mg/kg, the mean Cmax in the brain was 179 and 686 nM of minzasolmin (see Table 1 in ref. 1). Due to its short half-life in the brain of 0.56 to 0.60 h, the respective mean AUCs_0-6h_, namely 220 or 926 h*nM at 1 and 5 mg/kg, were not much larger than the Cmax. Barely any drug was left in the brain within a few hours after administration (i.e., ~0.35 or ~7 nM at 6 h after administration of 1 or 5 mg/kg of minzasolmin, when extrapolated from the measured plasma concentrations at that time point and applying the reported brain/plasma ratio of 0.3; these brain concentrations at 6 h were presumably below the limit of detection as they are not shown in Suppl. Fig. 1 in ref. 1). The authors also measured minzasolmin concentrations in the Thy1-aSyn animals at the end of the study and detected total brain concentrations of 28 and 163 nM at 1 h post-dosing of 1 and 5 mg/kg, respectively (Table 2 in ref. 1). This is about 70% less than the levels that were expected from the preparatory PK study in wild-type mice (compare brain concentrations at 1 h in Suppl. Fig. 1 in ref. 1) and suggests an even lower overall exposure of minzasolmin in the mixed C57BL/6 genetic background Thy1-aSyn mice as compared to the C57BL/6 wild-type mice.

The unbound, free drug concentration is usually deemed the one that is pharmacologically active^4,5^. For minzasolmin, actual values for the mouse or estimation from potential in vitro brain homogenate binding experiments were not publicly reported. However, the unbound fraction in human plasma is ~1%^6^, indicating that the largest portion of minzasolmin is protein- and/or lipid-bound. Assuming cerebrospinal fluid (CSF) being a surrogate for brain extracellular fluid, one may estimate from the reported CSF to unbound plasma concentration ratio of ~0.75 in human^6^ that the unbound drug concentration in the brain is comparable to the unbound plasma concentration. Presuming the situation is similar in mice, only a tiny fraction (~1 %; i.e., 0.3 or 1. 6 nM at 1 h after the last oral treatment of 1 and 5 mg/kg in Thy1-aSyn mice) of the total, measured drug concentration in the brain is available to act on its target.

One immediate question that emerges is why a drug like minzasolmin with such a short half-life in mice was injected only once and not twice a day to maintain a more constant exposure over 24 h? Alternatively, one could have investigated other means of drug delivery, such as drug-food admix, which is known to provide more constant drug levels over time. It is plausible that the authors may have considered such alternatives but missed to report them. Another question that arises concerns the treatment regimen of minzasolmin, which was administered only 5 days per week (Monday to Friday) during the chronic treatment period, representing roughly 70% of all treatment days. Such an interrupted treatment schedule is unusual for chronic preclinical pharmacology studies, and no rationale was provided for it. I wonder whether the authors opted for this regimen to reduce the overall cost of the study by minimizing weekend work or because the Thy1-aSyn mice are too fragile to support daily IP injections? A last question relates to the dose and as to why it was not increased to boost overall drug exposure? Is it possible that concerns for toxicity at higher doses prevented such an approach?

Irrespective of these procedural considerations, the main question left to answer is how minzasolmin was able to down-regulate aSyn levels in the brain of Thy1-aSyn mice and exert robust behavioral effects^1^, given the minimal time per day it was available to act on its target?

The molecular mechanism by which minzasolmin works has not yet been fully resolved. If UCB0599 was truly “binding and stabilizing unfolded alpha-synuclein” (which supposedly refers to monomers) as proposed in the Discussion of the UCB paper by ref. 6, one would, in return, expect that large drug concentrations were needed for it to be effective because aSyn is an abundant protein^7^. This becomes apparent in the example that the authors quoted themselves in the Discussion when they compared UCB0599 with the use of the transthyretin stabilizer tafamidis for treating hereditary transthyretin amyloidosis. Tafamidis, in fact needs to be given at large micromolar plasma concentrations to be able to bind to sufficient transthyretin proteins to be clinically efficacious^8,9^. Recent findings from a structural in vitro investigation^10^, however, suggest that minzasolmin rather binds to aSyn oligomers, thereby altering their rigidity and membrane interactions, and as a consequence, reduce the likelihood of oligomers seeding larger fibrils. Acting on aSyn oligomers rather than monomers would potentially reduce the requirement for the actual amount of drug that needs to be present to interfere with the aSyn aggregation process. Still, how could an only temporary (few hours/day; only 5 days a week) interference with membrane-bound aSyn account for slowing down the disease process in Thy1-aSyn mice^1^? As the kinetics of seeding, fibrillization, and aggregation of aSyn can be exponential (at least in vitro), temporary interference might be enough to slow down that process sufficiently. However, how would this then explain that UCB0599 reduced total aSyn levels in cortex and hippocampus in a dose-dependent manner, but dose-dependency was not observed in the reduction of proteinase-K resistant aSyn aggregates^1^?

Because of the uncertainty around the exact mechanism of action of minzasolmin and the technical difficulties of determining its supposed interaction with membrane-bound aSyn in a relevant model system, I believe that there is no public information on the actual potency of the drug available. This presents a challenge in establishing proper pharmacokinetic/pharmacodynamic (PK/PD) effect relationships for minzasolmin in Thy1-aSyn mice and makes me wonder how the dose for the ongoing clinical trial^2,3^ was selected. From the available public information, I speculate that the human doses of 90 and 180 mg bid were perhaps chosen to achieve similar Cmax levels^6^ as those observed in the mouse model with 1 and 5 mg/kg IP. However, minzasolmin is more stable and has a longer half-life in PD patients (~11–13 h)^6^, and accordingly, the AUC_1-12h_ achieved in human^6^ at the clinically tested doses are significantly larger than the corresponding AUCs that were achieved in the Thy1-aSyn mice.

What else could explain the efficacy of minzasolmin in mice? Interestingly, a prior study conducted by ref. 11 on the racemic form of minzasolmin, NPT200-11, in the identical mouse model also showed efficacy with the same dosing regimen. Here, the authors themselves noted the surprising efficacy despite the short half-life of NPT200-11 and proposed that perhaps a long-lived and biologically active metabolite could have been responsible, but no evidence was provided to support this. The absence or very low concentrations of the desmethyl and N-oxide metabolites found in the CSF of humans^6^ suggests that this explanation is unlikely unless there are species-specific metabolites present in mice that do not exist in humans.

In conclusion, I believe that additional clarifications from the authors regarding the questions raised above would be important to advance the discourse about how minzasolmin exerts its effects on Thy1-aSyn mice. It would also be important that other groups conduct independent efficacy studies with minzasolmin in Thy1-aSyn mice and other in vitro and in vivo PD models to gain better insight into its mode of action and understand the PK/PD relationship in animals.
